# The clinical value of nutritional and inflammatory indicators in predicting pneumonia among patients with intracerebral hemorrhage

**DOI:** 10.1038/s41598-024-67227-y

**Published:** 2024-07-13

**Authors:** Guang Zhao, Yuyang Chen, Yuting Gu, Xiaohua Xia

**Affiliations:** 1 Department of Emergency Medicine, The First People’s Hospital of Kunshan, Kunshan, 215300 Jiangsu China; 2 Jiangsu University Health Science Center, Kunshan, Jiangsu China

**Keywords:** ICH, SAP, CONUT, In-hospital death, MIMIC-IV, Diseases, Predictive markers, Prognostic markers

## Abstract

Immunosuppression and malnutrition play pivotal roles in the complications of intracerebral hemorrhage (ICH) and are intricately linked to the development of stroke-associated pneumonia (SAP). Inflammatory markers, including NLR (neutrophil-to-lymphocyte ratio), SII (systemic immune inflammation index), SIRI (systemic inflammatory response index), and SIS (systemic inflammation score), along with nutritional indexes such as CONUT (controlling nutritional status) and PNI (prognostic nutritional index), are crucial indicators influencing the inflammatory state following ICH. In this study, our objective was to compare the predictive efficacy of inflammatory and nutritional indices for SAP in ICH patients, aiming to determine and explore their clinical utility in early pneumonia detection. Patients with severe ICH requiring ICU admission were screened from the Medical Information Mart for Intensive Care IV (MIMIC-IV) database. The outcomes included the occurrence of SAP and in-hospital death. Receiver operating characteristic (ROC) analysis, multivariate logistic regression, smooth curve analysis, and stratified analysis were employed to investigate the relationship between the CONUT index and the clinical outcomes of patients with severe ICH. A total of 348 patients were enrolled in the study. The incidence of SAP was 21.3%, and the in-hospital mortality rate was 17.0%. Among these indicators, multiple regression analysis revealed that CONUT, PNI, and SIRI were independently associated with SAP. Further ROC curve analysis demonstrated that CONUT (AUC 0.6743, 95% CI 0.6079–0.7408) exhibited the most robust predictive ability for SAP in patients with ICH. Threshold analysis revealed that when CONUT < 6, an increase of 1 point in CONUT was associated with a 1.39 times higher risk of SAP. Similarly, our findings indicate that CONUT has the potential to predict the prognosis of patients with ICH. Among the inflammatory and nutritional markers, CONUT stands out as the most reliable predictor of SAP in patients with ICH. Additionally, it proves to be a valuable indicator for assessing the prognosis of patients with ICH.

## Introduction

Intracerebral hemorrhage (ICH), as a subtype of stroke, is marked by elevated mortality and a heightened incidence of disability^1^. While the mortality rate of stroke is declining, the clinical ramifications of stroke remain a focal point of concern. Stroke-associated pneumonia (SAP), a prevalent complication of stroke, significantly impedes patients' neurological recovery and exacerbates mortality rates, thereby extending hospitalization durations and imposing substantial economic strains on healthcare systems^2^. The precise identification of risk factors for SAP in individuals with ICH, along with prompt interventions, holds paramount importance for optimizing patient prognosis.

Research indicates that the compromised immune function resulting from ICH renders patients more vulnerable to infections, with immunosuppression emerging as a significant mechanism underlying SAP^3^. Consequently, biomarkers implicated in immune alterations and systemic inflammatory responses may play a role in SAP development. Studies have linked parameters such as white blood cell count, neutrophil count, lymphocyte count, as well as inflammatory biomarkers like the monocyte-to-lymphocyte ratio and the C-reactive protein albumin ratio, to adverse outcomes in SAP episodes and stroke^4,5^. Malnutrition is common among patients with ICH and is linked to higher risks of all-cause mortality and unfavorable functional outcomes^6^. Existing malnutrition can worsen during an ICH episode, potentially heightening the risk of adverse outcomes for the patient^7^. Hence, malnutrition may serve as a predisposing factor for SAP in individuals with ICH. Research has shown that biological indicators can reliably detect the onset of associated diseases, resulting in significant time and cost savings^8,9^. Additionally, they furnish medical personnel with valuable insights for precise diagnosis and treatment planning.

Based on these, the study aimed to assess the predictive capacity of inflammatory biomarkers (NLR, SII, SIRI, SIS) and nutritional indexes (CONUT, PNI) for predicting SAP occurrence in patients with ICH. The investigation sought to elucidate the potential usefulness of these biomarkers in early SAP identification, providing a comprehensive understanding of their predictive value in the context of ICH. Additionally, the study aimed to pinpoint the most effective predictors and examine their correlation with SAP.

## Methods

### Study population

**Figure 1:**
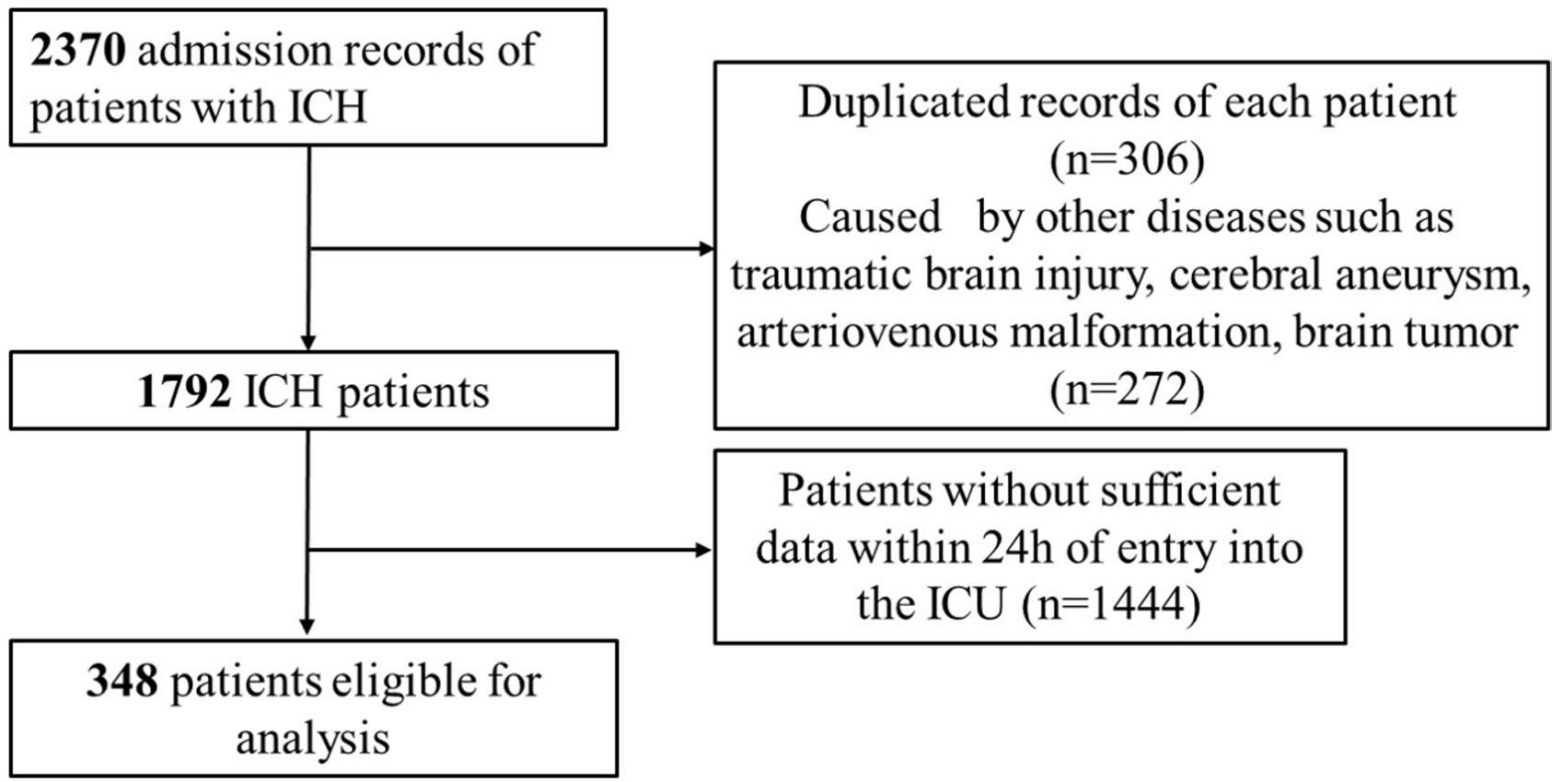
The flowchart of the study.

The current investigation utilized health-related data from the MIMIC-IV (version 2.2) repository, a widely-used extensive database developed and overseen by the MIT computational physiology laboratory^10^. Guang Zhao, a researcher, adhered to all access requirements and conducted the data extraction. This study included patients diagnosed with ICH based on the International Classification of Diseases, 9th and 10th Revision^11^. Specific exclusion criteria were applied as follows: (1) individuals under the age of 18 upon their initial admission; (2) patients with ICH caused by traumatic brain injury, brain tumor, cerebral aneurysm, or cerebral arteriovenous malformation; (3) patients with multiple admissions for ICH, with data extracted only from the first admission; (4) patients with severe liver and kidney conditions, leukemia, lymphoma, other hematological diseases, or malignant tumors; (5) patients lacking sufficient data, specifically Lymphocyte, serum albumin and cholesterol. In total, 348 patients were enrolled in this research (Fig. 1).

### Data collection

The information extraction process utilized PostgreSQL (version 14.10) software and Navicat Premium (version 16), with Structured Query Language (SQL) being the tool for extraction. The potential variables were grouped into four main categories: (1) demographic variables, including age, race, sex, and weight. (2) Complications, encompassing hypertension, diabetes, hyperlipemia, cognitive deficits, and delirium. (3) Laboratory indicators, consisting of RBC, Hb, PLT, WBC, neutrophil, lymphocyte, monocyte, serum glucose, albumin, serum sodium, serum potassium, serum creatinine, HDL, LDL, TC, and TG. (4) The Glasgow Coma scale score (GCS), patients with ICH were stratified into three groups based on GCS scores: 13–15 for mild coma, 9–12 for moderate coma, and 3–8 for severe coma. (5) Equations were applied to calculate various parameters. *Inflammation index*: NLR = count of neutrophils/count of lymphocytes; LMR = count of lymphocytes/count of monocytes; SIRI = (count of neutrophils × count of monocytes)/count of lymphocytes; SII = (count of neutrophils × count of platelets)/count of lymphocytes; Systemic inflammation score (SIS): based on lymphocyte to monocyte ratio and serum albumin level; Lymphocyte to monocyte ratio > 2.96 and serum albumin > 40.00 g/L was 0 points; One point was defined as lymphocyte-to-monocyte ratio ≤ 2.96 or serum albumin ≤ 40.00 g/L; Two points were defined as lymphocyte-to-monocyte ratio ≤ 2.96 and serum albumin ≤ 40.00 g/L^12^; *Nutritional index*: The controlling nutritional status (CONUT) score was calculated from the serum albumin level, peripheral blood lymphocyte count and total cholesterol level of the patients, and the values of each index were assigned according to the laboratory test results^13^. (1) Serum albumin level ≥ 35 g/L, 0 points, 30–34 g/L, 2 points, 25–29 g/L, 4 points, < 25 g/L, 6 points; (2) Lymphocyte ≥ 1.6 × 10^9^/L, 0 points, (1.2–1.6) × 10^9^/L,1 point, (0.8–1.1) × 10^9^/L, 2 points, < 0.8 × 10^9^/L, 3 points; (3) Total cholesterol ≥ 180 mg/dL, 0 point, 140–179 mg/dL, 1 point, 100–139 mg/dL, 2 points, < 100 mg/dL, 3 points. The scores of the above three indicators were summed to obtain a total score (range, 0–12), with higher total scores indicating worse nutritional status. Prognostic Nutritional Index (PNI) = serum albumin level + 5 × peripheral blood lymphocyte count. All laboratory variables and disease severity scores were obtained from data generated within 24 h of patient entry into the ICU.

### Statistical analysis

Continuous variables were presented as means ± standard deviations (SDs) or medians (interquartile ranges, IQRs) and compared using Student’s *t*-test or Kruskal–Wallis rank sum test, as appropriate. Categorical variables were expressed as frequencies and percentages, and Fisher exact tests were employed for comparison when appropriate. Logistic regression analysis was used to evaluate the independent correlation between NLR/LMR/SIRI/SII/SIS/PNI/CONUT and SAP or in-hospital mortality^14^. An extended logistic model approach was applied for different covariates-adjusted models, leading to the construction of three models: Model 1, adjusted for age, race, sex, marital status, and weight; Model 2, additionally adjusted for hypertension, diabetes, hyperlipidemia, cognitive deficits, and delirium. The presence of confusion was evaluated by including or excluding covariates in the logistic regression model, and regression coefficients were compared. A smoothing function logistics regression model was used for threshold analysis of the correlation between CONUT and study outcomes^15^. Threshold levels (i.e. inflection points) were determined with the use of likelihood ratio tests and bootstrap resampling. The predictive ability of NLR/SII/SIRI/PNI/CONUT was determined by calculating the area under the receiver operating characteristic curve (AUC). Receiver operating characteristic (ROC) analyses were utilized to evaluate predictive performance. Stratified analyses were conducted to assess potential modifications of the association between CONUT and SAP in ICH patients. A significance level of two-sided P values < 0.05 was considered statistically significant. Statistical analyses were conducted using IBM SPSS 24.0 (https://www.ibm.com/analytics/spss-statistics-software) and Empowerstats software (http://www.empowerstats.net/cn/index.php).

### Ethics statement

This study adhered to the regulations outlined in the Helsinki Declaration. Approval for accessing the MIMIC-IV database was granted by the review committee of Beth Israel Deaconess Medical Center and Massachusetts Institute of Technology. As the data is openly accessible within the MIMIC-IV database, and to safeguard patient privacy, all personal data underwent de-identification, with only randomized codes assigned for patient identification. Consequently, the Ethical Committee of the First People's Hospital of Kunshan, China, waived the necessity for informed consent and ethical approval.

## Results

### Baseline characteristics

This study enrolled a total of 348 ICU patients diagnosed with ICH. The average age of the participants was 70.8 years, with 181 individuals (52.01%) being male. Among the entire sample, 74 patients (21.3%) developed SAP, and 59 patients (17.0%) experienced mortality during their hospitalization. The baseline characteristics of patients with ICH are presented in Table 1. Patients with SAP were found to be older and had an extended length of stay (LOS) in the ICU (p < 0.001). They also exhibited a higher rate of endotracheal intubation (p < 0.001) and presented with elevated white blood cell count (p = 0.01), increased neutrophil count (p = 0.028), higher blood glucose level (p < 0.001), lower albumin level (p = 0.011), and reduced cholesterol level (p = 0.004). These findings suggest a severe inflammatory response, poor nutritional status, and a higher likelihood of immunosuppression in SAP patients. The biomarker levels exhibited notable distinctions between the stroke-associated pneumonia (SAP) and non-SAP groups. In the SAP group, NLR (p < 0.001), SII (p = 0.041), SIRI (p < 0.001), SIS (p = 0.031), and CONUT (p < 0.001) were significantly elevated, while LMR (p < 0.001) and PNI (p = 0.001) were significantly lower compared to the non-SAP group. Within the non-survival group, NLR (p < 0.001), SII (p = 0.014), SIRI (p = 0.003), and CONUT (p < 0.001) were markedly higher, while LMR (p = 0.024) was significantly lower in the SAP group compared to the non-SAP group. These findings underscore the potential relevance of these biomarkers to both the occurrence of SAP and its impact on survival outcomes.

**Table 1:** Baseline characteristics of the 348 patients with ICH.

	Total (n = 348)	SAP development			Death in hospital		
		Non-SAP (n = 274)	SAP (n = 74)	P-value	Survivor (n = 289)	Non-survivor (n = 59)	P-value
Demographics							
Age (years)	70.80 ± 14.49	70.61 ± 14.63	71.51 ± 14.05	0.732	70.07 ± 14.76	74.40 ± 12.62	**0.042**
Weight	79.60 ± 24.52	79.11 ± 24.42	81.42 ± 24.94	0.653	79.07 ± 24.41	82.22 ± 25.07	0.138
Marital status				**0.045**			0.247
Single	65 (18.68%)	56 (20.44%)	9 (12.16%)		57 (19.72%)	8 (13.56%)	
Married	146 (41.95%)	117 (42.70%)	29 (39.19%)		123 (42.56%)	23 (38.98%)	
Divorced	20 (5.75%)	18 (6.57%)	2(2.70%)		18 (6.23%)	2 (3.39%)	
Widowed	117 (33.62%)	83 (30.29%)	34 (45.95%)		91 (31.49%)	26 (44.07%)	
Race				**0.023**			0.216
White	206 (59.20%)	169 (61.68%)	37 (50.00%)		177 (61.25%)	29 (49.15%)	
Black	36 (10.34%)	31 (11.31%)	5 (6.76%)		29 (10.03%)	7 (11.86%)	
Others	106 (30.46%)	74 (27.01%)	32 (43.24%)		83 (28.72%)	23 (38.98%)	
Gender				**0.006**			0.707
Male	181 (52.01%)	132 (48.18%)	49 (66.22%)		149 (51.56%)	32 (54.24%)	
Female	167 (47.99%)	142 (51.82%)	25 (33.78%)		140 (48.44%)	27 (45.76%)	
Clinical features							
RBC (m/uL)	4.19 (3.71–4.62)	4.23 (3.79–4.65)	4.03 (3.54–4.55)	0.051	4.19 (3.73–4.62)	4.22 (3.64–4.69)	0.802
PLT (K/uL)	213.50 (166.00–252.00)	217.00 (173.75–255.00)	190.00 (148.50–241.00)	**0.004**	215.00 (170.00–251.25)	191.00 (145.50–256.00)	0.136
WBC (K/uL)	9.80 (7.70–13.00)	9.60 (7.50–12.40)	11.25 (8.35–14.10)	**0.01**	9.40 (7.45–12.25)	12.30 (9.83–14.95)	** < 0.001**
Neutrophil (K/uL)	7.66 (5.16–10.61)	7.31 (5.10–10.30)	8.84 (5.80–11.96)	**0.028**	7.44 (5.13–10.15)	9.51 (6.47–13.06)	**0.006**
Lymphocyte (K/uL)	1.23 (0.83–1.81)	1.29 (0.87–1.87)	1.07(0.67–1.42)	**0.005**	1.25 (0.88–1.84)	0.90 (0.62–1.73)	**0.039**
Monocyte (K/uL)	0.67(0.52–0.94)	0.65 (0.50–0.89)	0.76(0.61–1.06)	**0.004**	0.68 (0.52–0.94)	0.64 (0.50–0.93)	0.819
Glucose (mg/dL)	124.00 (108.00–148.55)	119.36 (104.44–144.88)	135.54 (122.11–161.19)	** < 0.001**	118.68 (104.67–138.37)	151.20 (134.04–180.54)	** < 0.001**
Albumin (mg/dL)	39.00 (36.00–43.00)	39.00 (36.25–43.00)	37.00 (33.25–41.00)	**0.011**	39.00 (36.00–43.00)	38.00 (34.00–42.50)	0.172
Lactate(mmol/L)	1.80(1.20–2.30)	1.70 (1.20–2.20)	1.95 (1.10–2.33)	0.397	1.60 (1.20–2.20)	2.20 (1.60–3.20)	** < 0.001**
HDL (mg/dL)	51.00 (40.00–63.00)	52.00 (42.00–66.00)	44.00 (35.00–54.00)	** < 0.001**	51.00 (40.00–63.75)	47.00 (40.50–63.00)	0.469
LDL (mg/dL)	89.50 (66.00–116.00)	94.00 (68.00–118.25)	80.50 (55.25–109.50)	**0.016**	94.00 (68.00–119.00)	71.00 (56.50–105.00)	**0.004**
TC (mg/dL)	166.00 (134.50–196.00)	170.00 (140.00–197.00)	152.00 (124.50–180.75)	**0.004**	169.00 (139.00–197.00)	153.00 (122.50–183.00)	**0.019**
TG (mg/dL)	96.00 (72.00–134.25)	94.00 (72.00–128.00)	117.00 (74.25–149.75)	**0.016**	95.00 (72.00–131.00)	104.00 (71.50–153.50)	0.359
NLR	6.37 (3.54–11.39)	5.84 (3.21–10.09)	9.71 (4.84–15.06)	** < 0.001**	5.83 (3.36–10.67)	8.67 (5.78–15.31)	** < 0.001**
LMR	1.86 (1.09–2.69)	1.95 (1.18–2.88)	1.34 (0.77–2.06)	** < 0.001**	1.92 (1.16–2.76)	1.55 (0.78–2.40)	**0.024**
SII	1247.10 (686.47–2309.51)	1154.10 (664.80–2205.42)	1546.68 (906.90–2704.16)	**0.041**	1169.81 (661.27–2136.76)	1830.49 (1020.71–2851.01)	**0.014**
SIRI	4.13 (2.17–8.68)	3.49 (1.92–7.19)	6.72 (3.40–13.57)	** < 0.001**	3.79 (1.98–7.57)	7.18 (2.98–14.33)	**0.003**
PNI	45.87 (41.19–50.43)	46.35 (42.01–50.79)	43.10 (37.21–48.49)	**0.001**	46.15 (41.70–50.65)	43.65 (37.77–49.93)	0.073
SIS				**0.031**			0.724
0	40 (11.49%)	35 (12.77%)	5 (6.76%)		35 (12.77%)	5 (6.76%)	
1	124 (35.63%)	104 (37.96%)	20 (27.03%)		104 (37.96%)	20 (27.03%)	
2	184 (52.87%)	135 (49.27%)	49 (66.22%)		135 (49.27%)	49 (66.22%)	
CONUT	2.00 (1.00–4.00)	2.00 (1.00–3.00)	3.00 (2.00–6.00)	** < 0.001**	2.00 (1.00–4.00)	3.00 (2.00–5.00)	** < 0.001**
GCS				0.475			0.918
Mild	302 (86.78%)	240 (87.59%)	62 (83.78%)		251 (86.85%)	51 (86.44%)	
Moderate	38 (10.92%)	29 (10.58%)	9 (12.16%)		31 (10.73%)	7 (11.86%)	
Severe	8 (2.30%)	5 (1.82%)	3(4.05%)		7 (2.42%)	1 (1.69%)	
Complications							
Hypertension				0.45			0.496
No	128 (36.78%)	98 (35.77%)	30 (40.54%)		104 (35.99%)	24 (40.68%)	
Yes	220 (63.22%)	176 (64.23%)	44 (59.46%)		185 (64.01%)	35 (59.32%)	
Diabetes				0.939			0.336
No	248 (71.26%)	195 (71.17%)	53 (71.62%)		209 (72.32%)	39 (66.10%)	
Yes	100 (28.74%)	79 (28.83%)	21 (28.38%)		80 (27.68%)	20 (33.90%)	
Hyperlipidemia				0.616			0.387
No	183 (52.59%)	146 (53.28%)	37 (50.00%)		155 (53.63%)	28 (47.46%)	
Yes	165 (47.41%)	128 (46.72%)	37 (50.00%)		134 (46.37%)	31 (52.54%)	
Cognitive deficits				0.187			0.318
No	334 (95.98%)	261 (95.26%)	73 (98.65%)		276 (95.50%)	58 (98.31%)	
Yes	14 (4.02%)	13 (4.74%)	1(1.35%)		13 (4.50%)	1 (1.69%)	
Delirium				0.366			0.31
No	326 (93.68%)	255 (93.07%)	71 (95.95%)		269(93.08%)	57 (96.61%)	
Yes	22 (6.32%)	19 (6.93%)	3(4.05%)		20 (6.92%)	2 (3.39%)	
Treatment							
Ventilation status				** < 0.001**			** < 0.001**
None	230 (66.09%)	216 (78.83%)	14 (18.92%)		213 (73.70%)	17 (28.81%)	
Oxygen	36 (10.34%)	21 (7.66%)	15 (20.27%)		31 (10.73%)	5 (8.47%)	
Tracheostomy	1 (0.29%)	0 (0.00%)	1 (1.35%)		1 (0.35%)	0 (0.00%)	
Endotracheal intubation	81 (23.28%)	37 (13.50%)	44 (59.46%)		44 (15.22%)	37 (62.71%)	
LOS for ICU	4.51 (2.28–7.78)	3.39 (1.94–5.78)	11.37 (6.15–17.57)	** < 0.001**	4.52 (2.32–7.75)	4.17 (1.96–7.91)	0.587

*ICH* intracerebral hemorrhage, *WBC* white blood cell, *RBC* red blood cell, *PLT* platelets, *GCS* Glasgow coma scale, *NLR* neutrophil-to-lymphocyte ratio, *SII* systemic immune-inflammation index, *SIRI* systemic inflammation response index, *SIS* systemic inflammation score, *CONUT* the controlling nutritional status, *PNI* prognostic nutritional index.

The P values < 0.05 are written in bold text.

### Relationship between CONUT and clinical outcome

Multivariable logistic regression analyses were conducted to formulate three models (Table 2): an unadjusted model, a partially adjusted model (adjusted for age, sex, weight, race, and marital status), and a fully adjusted model (adjusted for age, sex, weight, race, marriage, hypertension, diabetes, hyperlipidemia, cognitive deficits, and delirium). In the three models, with SAP as the outcome variable, the findings indicated that CONUT, PNI, and SIRI remained statistically significant even after adjusting for other confounding factors. Elevated NLR and SII were identified as risk factors in the unadjusted model. When in-hospital mortality was considered as the outcome variable, CONUT, NLR, SIRI, and SII retained significance even after adjusting for other confounding factors. These results underscore the robustness of CONUT, NLR, SIRI, and SII as potential predictors, demonstrating their independent associations with the outcomes of interest, and underscoring the importance of these inflammatory and nutritional markers in predicting adverse outcomes in patients with ICH.

**Table 2:** Logistic regression models for SAP occurrence and in-hospital mortality.

Variable	Crude	P value	Model I	P value	Model II	P value
	HR (95% CI)		HR (95% CI)		HR (95% CI)	
SAP occurrence						
CONUT	1.273 (1.142, 1.420)	**0.00001**	1.243 (1.107, 1.395)	**0.00023**	1.227 (1.090, 1.381)	**0.00069**
PNI	0.956 (0.926, 0.988)	**0.00715**	0.962 (0.929, 0.996)	**0.02703**	0.965 (0.931, 0.999)	**0.04605**
SIS						
0	1 (ref)		1 (ref)		1 (ref)	
1	1.346 (0.470, 3.855)	0.57977	1.278 (0.429, 3.802)	0.65964	1.246 (0.419, 3.705)	0.6929
2	2.541 (0.942, 6.854)	0.06554	2.443 (0.859, 6.942)	0.09376	2.318 (0.812, 6.612)	0.11602
NLR	1.028 (1.003, 1.053)	**0.02816**	1.025 (0.998, 1.052)	0.06552	1.025 (0.998, 1.052)	0.06562
LMR	0.763 (0.610, 0.955)	**0.01825**	0.804 (0.636, 1.015)	0.06686	0.819 (0.648, 1.036)	0.09548
SIRI	1.039 (1.013, 1.065)	**0.0033**	1.038 (1.010, 1.067)	**0.00829**	1.037 (1.008, 1.066)	**0.01085**
SII	1.000 (1.000, 1.000)	0.45752	1.000 (1.000, 1.000)	0.48122	1.000 (1.000, 1.000)	0.44351
WBC	1.080 (1.018, 1.145)	**0.01089**	1.074 (1.009, 1.144)	**0.02574**	1.075 (1.009, 1.145)	**0.02566**
Lactate	1.146 (0.883, 1.488)	0.30412	1.159 (0.872, 1.540)	0.3108	1.127 (0.845, 1.504)	0.41694
Glucose	1.007 (1.001, 1.012)	**0.01179**	1.006 (1.000, 1.011)	**0.0336**	1.009 (1.003, 1.016)	**0.00608**
Albumin	0.946 (0.907, 0.986)	**0.00953**	0.951 (0.909, 0.994)	**0.02771**	0.953 (0.910, 0.998)	**0.0411**
Ventilation status						
None	1 (ref)		1 (ref)		1 (ref)	
Oxygen	11.020 (4.686, 25.916)	** < 0.00001**	10.600 (4.316, 26.033)	** < 0.00001**	11.262 (4.479, 28.317)	** < 0.00001**
Endotracheal intubation	18.347 (9.155, 36.771)	** < 0.00001**	19.112 (9.130, 40.011)	** < 0.00001**	20.303 (9.416, 43.780)	** < 0.00001**
Death of hospital						
CONUT	1.209 (1.080, 1.355)	**0.00103**	1.194 (1.058, 1.348)	**0.0041**	1.185 (1.047, 1.341)	**0.00739**
PNI	0.978 (0.945, 1.013)	0.21462	0.986 (0.951, 1.024)	0.47051	0.989 (0.952, 1.027)	0.55969
SIS						
0	1 (ref)		1 (ref)		1 (ref)	
1	1.025 (0.379, 2.776)	0.96064	1.080 (0.384, 3.035)	0.88378	1.049 (0.372, 2.959)	0.92773
2	1.284 (0.500, 3.303)	0.60339	1.129 (0.415, 3.071)	0.81224	1.064 (0.388, 2.922)	0.90382
NLR	1.035 (1.009, 1.062)	**0.00855**	1.042 (1.014, 1.070)	**0.00306**	1.043 (1.014, 1.072)	**0.00305**
LMR	0.910 (0.742, 1.115)	0.36252	0.934 (0.760, 1.148)	0.51736	0.953 (0.771, 1.177)	0.65187
SIRI	1.033 (1.007, 1.059)	**0.01285**	1.038 (1.009, 1.067)	**0.00892**	1.037 (1.008, 1.067)	**0.01108**
SII	1.000 (1.000, 1.000)	0.10555	1.000 (1.000, 1.000)	**0.03134**	1.000 (1.000, 1.000)	**0.03258**
WBC	1.158 (1.085, 1.235)	** < 0.00001**	1.190 (1.108, 1.279)	** < 0.00001**	1.191 (1.108, 1.280)	** < 0.00001**
Lactate	1.704 (1.258, 2.307)	**0.00057**	1.693 (1.227, 2.337)	**0.00136**	1.643 (1.186, 2.277)	**0.00282**
Glucose	1.014 (1.008, 1.020)	** < 0.00001**	1.014 (1.008, 1.020)	** < 0.00001**	1.020 (1.012, 1.028)	** < 0.00001**
Albumin	0.969 (0.926, 1.014)	0.17458	0.982 (0.936, 1.031)	0.46196	0.985 (0.938, 1.035)	0.55192
Ventilation status						
None	1 (ref)		1 (ref)		1 (ref)	
Oxygen	2.021 (0.696, 5.868)	0.1958	1.706 (0.566, 5.139)	0.34233	1.763 (0.577, 5.382)	0.31953
Endotracheal intubation	10.536 (5.447, 20.378)	** < 0.00001**	11.771 (5.778, 23.980)	** < 0.00001**	12.836 (6.108, 26.972)	** < 0.00001**

Adjust model I adjust for: age, race, sex, marital status, and weight; Adjust model II adjust for: age, race, sex, marital status, weight, hypertension, diabetes, hyperlipidemia, cognitive deficits, and delirium.

Significant values are in bold.

**Figure 2:**
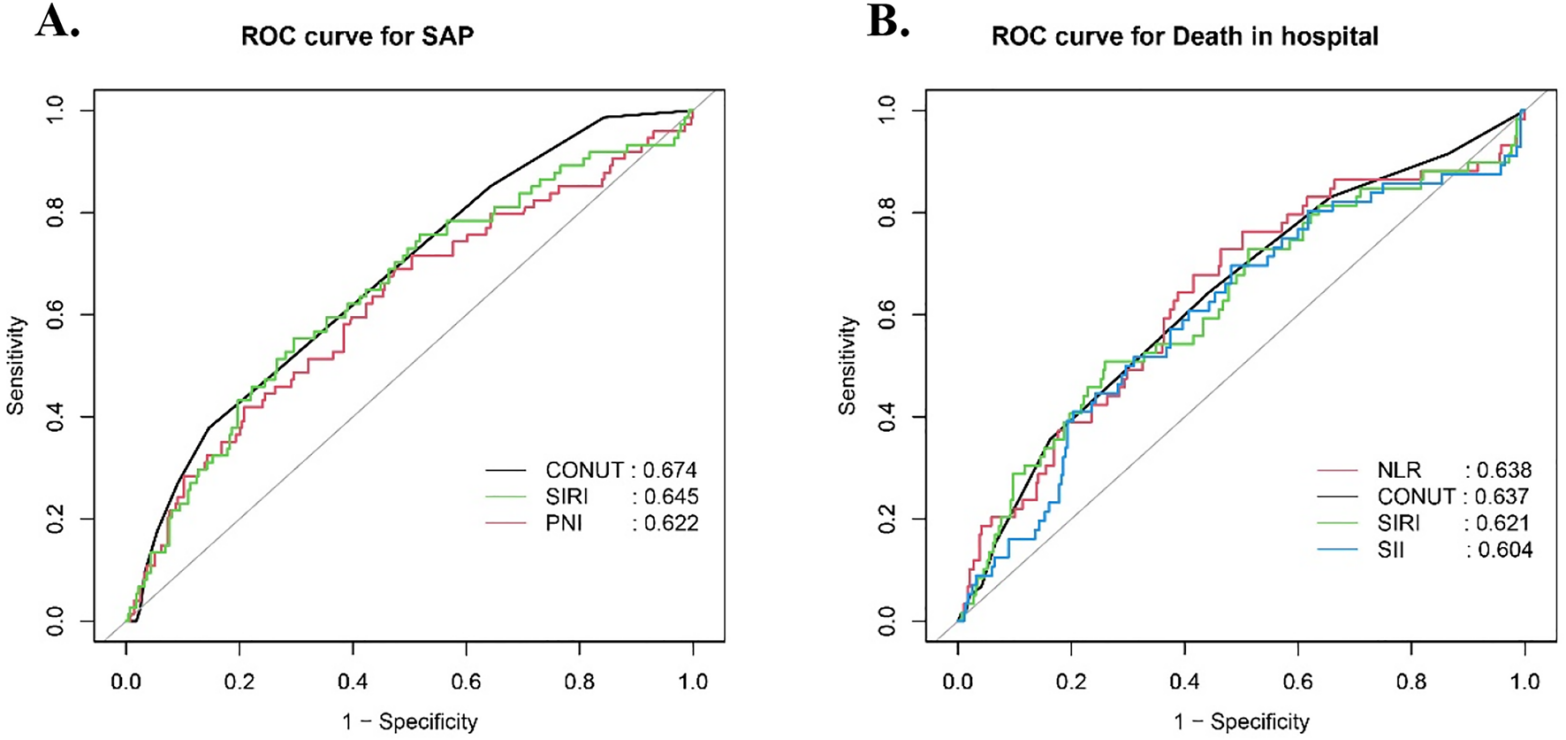
ROC curves of the CONUT, SIRI, and PNI for predicting SAP occurrence (**A**); ROC curves of the NLR, CONUT, SIRI, and SII for predicting death in hospital (**B**).

Based on the above results, ROC analysis was employed to assess the predictive ability of biomarkers for the occurrence of SAP and in-hospital mortality after ICH (Fig. 2). Regarding SAP as the outcome, CONUT (AUC 0.6743, 95% CI 0.6079–0.7408), PNI (AUC 0.6217, 95% CI 0.5462–0.6973), and SIRI (AUC 0.6449, 95% CI 0.5717–0.7181) were evaluated. The results indicate that CONUT exhibited a superior ability to predict SAP. When considering in-hospital mortality as the outcome, CONUT (AUC 0.6374, 95% CI 0.559–0.7158), NLR (AUC 0.6376, 95% CI 0.5558–0.7195), SIRI (AUC 0.6207, 95% CI 0.5361–0.7053), and SII (AUC 0.6041, 95% CI 0.5186–0.6896) were evaluated. This suggests that CONUT and NLR demonstrated equivalent predictive capabilities for in-hospital mortality. Details regarding the optimal cutoff, specificity, and sensitivity are outlined in Table 3.

**Table 3:** AUC in predicting the SAP occurrence and in-hospital mortality.

	AUC (95% CI)	Cutoff point	Specificity	Sensitivity	PPV	NPV
AUC in predicting SAP occurrence						
CONUT	0.6743 (0.6079–0.7408)	4.5	0.854	0.3784	0.4118	0.8357
PNI	0.6217 (0.5462–0.6973)	46.15	0.5255	0.6892	0.2818	0.8623
SIRI	0.6449 (0.5717–0.7181)	6.2286	0.7044	0.5541	0.3361	0.854
AUC in predicting death of hospital						
CONUT	0.6374 (0.559–0.7158)	2.5	0.5571	0.6441	0.2289	0.8846
NLR	0.6376 (0.5558–0.7195)	6.1325	0.5363	0.7288	0.2429	0.9064
SIRI	0.6207 (0.5361–0.7053)	7.1554	0.7405	0.5085	0.2857	0.8807
SII	0.6041 (0.5186–0.6896)	1199.1054	0.5179	0.6964	0.2241	0.8951

**Figure 3:**
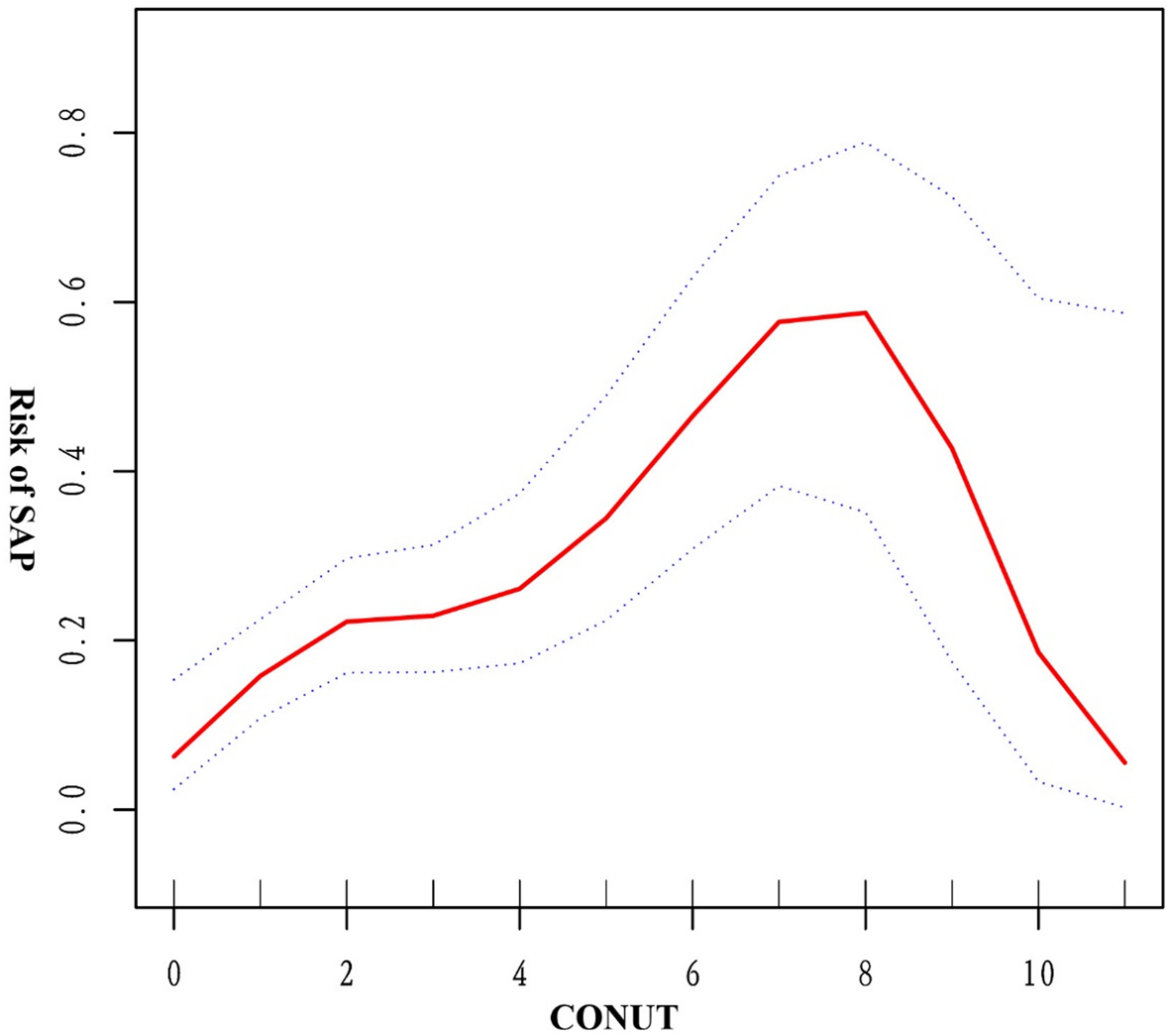
Smoothed curves of the associations between CONUT and risk of SAP in patients with ICH.

To delve deeper into the relationship between CONUT and SAP, we employed smooth curves to identify potential "inflection points" in the correlation. Surprisingly, the smooth curve depicted an inverted "U" shaped trend in the relationship between CONUT and SAP (Fig. 3, CONUT inflection point = 6). When CONUT < 6, the incidence of SAP increased by 1.398 times (p = 0.0001) for every 1-point increase in CONUT. Conversely, when CONUT > 6, the incidence of SAP increased by 0.846 times (Table4, p = 0.3906).

**Table 4:** Threshold analyses of CONUT on SAP occurrence for patients with ICH using logistic regression models.

	Crude β/OR (95% CI), P value	Adjusted β/OR (95% CI), P value
SAP		
CONUT < 6	1.450 (1.236, 1.702) < 0.0001	1.398 (1.177, 1.660) 0.0001
CONUT ≥ 6	0.871 (0.607, 1.248) 0.4507	0.846 (0.578, 1.239) 0.3906

Adjust model adjust for: age, sex, weight, race, marital status, hypertension, diabetes, hyperlipidemia, cognitive deficits, delirium and GCS.

### Stratified analyses by potential effect modifiers

**Figure 4:**
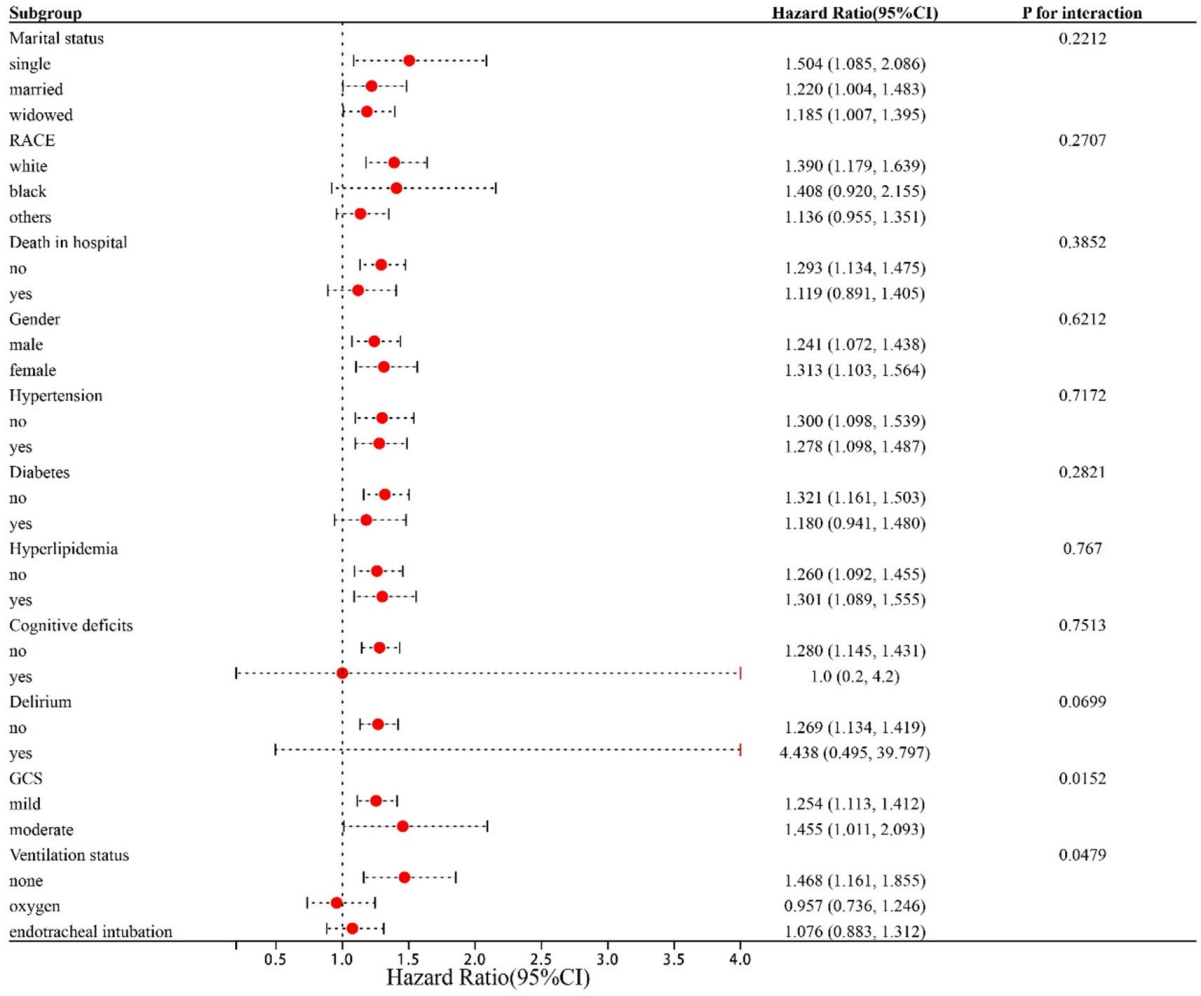
Forest plots of hazard ratios for the SAP occurrence in different subgroups. *HR* hazard ratio, *CI* confidence interval.

To assess the relationship between CONUT and the risk of SAP across various subgroups of patients with ICH, the study conducted further stratification (Fig. 4). The CONUT index demonstrated an association with a heightened risk of SAP within the ICH subgroup, particularly among those who were single, white, survivors, patients without delirium, and patients without cognitive deficits. Notably, within the GCS grouping, a P-value for interaction < 0.05 was observed. This finding suggests that a lower GCS score denotes poorer neurological function, diminished level of consciousness, severe ICH, higher likelihood of immunosuppression, and increased SAP incidence. Hence, the CONUT index alone may not suffice to predict SAP incidence.

## Discussion

SAP represents a prevalent and formidable complication, particularly in patients experiencing ICH^16^. This complication significantly exacerbates the patient's health status, extends the duration of hospitalization, and contributes to an elevated mortality rate. Research indicates that 16% of stroke patients experience acute lung injury within 36 h of admission, and 5–22% develop pneumonia during their hospital stay^17^. Our findings align with these patterns, revealing that SAP manifested in 74 (21.3%) of the studied patients. In contrast to ischemic stroke, there are few studies on SAP in ICH. In this prospective study, we conducted a novel comparison between inflammatory markers and nutritional indices to assess their predictive capacities for SAP and explore their clinical significance. The findings revealed that CONUT exhibited substantial predictive prowess for SAP, with a comparable performance NLR in predicting in-hospital mortality.

Most studies have associated SAP with stroke-induced immunodepression syndrome (SIDS)^18^. Throughout the progression of ICH, the inflammatory response and immune cells release cytokines, inducing the production of anti-inflammatory signals that impede infection and inflammation^19^. However, persistent inflammatory response will eventually exhaust the immune system, diminishing systemic immune activity, inhibiting systemic cellular immune responses, and precipitating a rapid reduction in lymphocytes, known as SIDS^20,21^. SIDS is a pivotal factor in SAP development, leading to diminished recognition and clearance of cytotoxic T lymphocytes, thereby increasing susceptibility^22–24^. In our study, we observed a notable decrease in lymphocyte counts within the SAP group compared to the non-SAP group. Lymphocytes, acting as the primary regulators of the body's immune system, hold a pivotal role in orchestrating host defense mechanisms against pathogens^25,26^. The diminished levels of lymphocytes observed in the SAP group may compromise immune functionality, thereby heightening susceptibility to SAP. Research has consistently demonstrated a significant correlation between heightened neutrophil levels and adverse outcomes such as early neurological deterioration, in-hospital mortality, and overall poor prognosis in patients with ICH^27^. Mounting evidence supports the association of an elevated NLR with factors like hematoma enlargement, perihematoma edema growth, and early neurological deterioration^25,28–30^. Notably, our investigation revealed markedly elevated neutrophil levels and NLR in the SAP group compared to the non-SAP group, underscoring the close relationship between NLR and the onset of SAP in ICH. It is pertinent to highlight the observation that both albumin and total cholesterol levels were notably lower in the SAP group compared to the non-SAP group. Existing literature underscores the significance of serum albumin in relation to systemic inflammatory response syndrome, aspiration pneumonia, and three-month mortality^31–33^. Low albumin levels are widely acknowledged as a reliable marker of malnutrition. Similarly, concerning total cholesterol, diminished levels are significantly linked to an augmented risk of ICH^34,35^. Building upon this insight, our study introduced CONUT, a nutritional indicator derived from serum albumin concentration, total lymphocyte count in peripheral blood, and total cholesterol concentration. Through multivariate logistic regression analysis, our study identified CONUT as an independent predictor of SAP occurrence in ICH patients. Particularly noteworthy was the observation that in patients with CONUT < 6, there was a notable upward trend in the risk of SAP associated with increasing CONUT levels. The ROC analysis further underscored the exceptional predictive prowess of CONUT for SAP, and demonstrated performance equivalence to NLR in forecasting in-hospital mortality. Notably, CONUT emerged as an indispensable factor in the comprehensive assessment of both SAP and mortality risk in ICH.

The CONUT, serving as an assessment index for body nutrition, is frequently employed as a valuable prognostic indicator for cardiovascular diseases, malignant tumors, and postoperative malnutrition^36–38^. Notably, our study unveiled an independent association between CONUT and SAP in cases of ICH. To delve deeper into the relationship between CONUT and SAP, we conducted a stratified analysis to assess the risk across various subgroups in ICH patients. Notably, within GCS subgroup, a significant p-value for interaction (< 0.05) underscores the pivotal role of GCS score in evaluating ICH severity. A lower GCS score correlates with more severe ICH, diminished neurological function, and increased susceptibility to immunosuppression^39^. While CONUT alone may not adequately predict SAP risk, it demonstrates clinical value in assessing the risk of in-hospital death. A similar limitation is observed in the subgroup of ventilation status, potentially attributed to the inherent risk elevation associated with endotracheal intubation. Despite these considerations, CONUT remains valuable for evaluating the risk of in-hospital death in patients with ICH. Research indicates that the swift and accurate identification of biomarkers and associated genes can not only significantly reduce time and economic costs but also offer invaluable insights for medical professionals to make precise diagnoses and treatment decisions^40–46^. Consequently, it is crucial to prioritize attention to CONUT in patients with ICH.

This study possesses several limitations. Firstly, the absence of information regarding the surgical treatment status of patients with intracerebral hemorrhage and the lack of personal history details, including smoking and alcohol consumption, are notable gaps. Secondly, the biomarkers were recorded only within 24 h after ICU admission. However, relevant biomarkers might undergo changes in the days following intracerebral hemorrhage onset. We recognize that utilizing theoretical modeling based on ordinary differential equations (ODE) has become pivotal in disease prediction^47–49^. Therefore, we plan to integrate ODE theory into our research to investigate changes in biomarkers at various time points post-onset and their predictive efficacy.

## Conclusion

In conclusion, our findings indicate that CONUT not only predicts the risk of SAP in patients with ICH but also serves as a valuable indicator for assessing the prognosis of patients with ICH.

## Data Availability

The data analyzed in this study were sourced from the Medical Information Mart for Intensive Care IV (MIMIC-IV) database (https://mimic.mit.edu/). Datasets produced and analyzed during this research are accessible from the corresponding author upon reasonable request.
